# A pilot clinical and radiographic study on the association between periodontitis and serious COVID‐19 infection

**DOI:** 10.1002/cre2.610

**Published:** 2022-08-06

**Authors:** Panagiotis Gardelis, Alkisti Zekeridou, Noemie Suh, Christophe Le Terrier, Andreas Stavropoulos, Catherine Giannopoulou

**Affiliations:** ^1^ Division of Regenerative Dental Medicine and Periodontology, University Clinics of Dental Medicine University of Geneva Geneva Switzerland; ^2^ Intensive Care Unit, Department of Acute Medicine University Hospitals of Geneva Geneva Switzerland; ^3^ Department of Periodontology, Faculty of Odontology Malmo University Malmo Sweden; ^4^ Division of Conservative Dentistry and Periodontology University Clinic of Dentistry, Medical University of Vienna Vienna Austria

**Keywords:** COVID‐19, intensive care unit, periodontal disease, SARS‐CoV‐2, severity

## Abstract

**Background:**

During the pandemic of COVID‐19, the scientific community tried to identify the risk factors that aggravate the viral infection. Oral health and specifically periodontitis have been shown to have a significant impact on overall health. Current, yet limited, evidence suggests a link between periodontal status and severity of COVID‐19 infection.

**Objectives:**

The present pilot study aimed to assess whether younger patients (≤60 years) that have been hospitalized in the intensive care unit (ICU) for severe COVID‐19 infection were susceptible to severe periodontitis.

**Material and Methods:**

All dentate patients ≤60 years of age diagnosed with COVID‐19 and surviving hospitalization in the ICU were considered for inclusion. Susceptibility to periodontitis was determined by assessing radiographic bone loss (RBL) in recent dental radiographs (posterior bitewings, periapical, and panoramic X‐rays). RBL in % was obtained from the most affected tooth and patients were classified into: Stage I, RBL ≤ 15%; Stage II, RBL = 15%−33% and Stage III/IV, RBL ≥ 33%. The grade was defined using the RBL to age ratio on the most severely affected tooth. Patients were attributed to: Grade A, ratio <0.25; Grade B, ratio 0.25−1 and Grade C, ratio >1. Patients classified into Stage III/IV and Grade C were considered highly susceptible to periodontitis.

**Results:**

Of 87 eligible patients, 30 patients were finally assessed radiographically and/or clinically; from the remaining 57 patients, 16 refused participation for various reasons and 41 could not be reached. Based on the radiographic assessment, all patients were periodontally compromised. Half of them were classified with Stage III/IV and Grade B or C; 26.7% were classified with Stage III/IV and Grade C.

**Conclusions:**

The present pilot study showed that about half of the patients suffering from severe forms of COVID‐19 infection in need of ICU admission suffered also from severe periodontitis, and about one‐fourth of them were highly susceptible to it.

## 1 INTRODUCTION

An outbreak of a new infectious viral disease influenced the whole world the last 3 years. The novel coronavirus SARS‐CoV‐2 appeared in late 2019 and has quickly spread over the earth (Liu et al., 2020). The scientific community put a lot of effort and investment to understand the mechanism of pathology and the risk factors of this violent virus infection that cost the life of a great number of people (Grasselli et al., 2020). Many people died especially in the first and second waves. Hyperinflammation (46.9%), septic shock (19.75%), multiorgan failure (16.05%), and cardiac arrest (8.64%) have been identified as the primary causes of COVID‐19‐related death (Du et al., 2020). Concomitant systemic diseases and the general status of the patients are continuously evaluated to identify predisposing/risk factors that may influence the severity and the mortality of COVID‐19. Among these, hypertension, obesity, and diabetes are the three comorbidities with the most unfavorable outcomes, which are hospitalization, intensive care unit (ICU) admission, and eventually death (Richardson et al., 2020; Zhou et al., 2020).

In this context, oral health has been shown to have a significant impact on overall health. Periodontitis, in particular, has been associated with a number of systemic diseases/conditions, such as diabetes, atherosclerotic heart disease, Alzheimer's disease, obesity, various cancers, etc. (Jepsen et al., 2015; Linden et al., 2013; Wu & Nakanishi, 2014). The main mechanisms for these associations seem to be the systemic release of locally produced proinflammatory cytokines in response to the infection in the periodontal pocket, and the systemic dissemination of microbial products, resulting in inflammation in distant organs (Hasturk & Kantarci, 2015). The majority of the population copes well with this low‐level infection and suffers only from mild/moderate forms of periodontal disease, with bone loss around the teeth that is not excessive and/or only a few teeth affected. A fraction of the population, however, is highly susceptible and develops severe forms of the disease, showing excessive bone loss to the majority of the teeth, and occasionally at a younger age. Tissue destruction in these patients is due to an exacerbation of inflammatory cytokines (e.g., interleukin [IL]‐1, IL‐6, tumor necrosis factor [TNF]‐α), which is disproportional to this, otherwise, mild chronic inflammation. This response appears to have similarities with what is observed in patients suffering from severe COVID‐19 infection (Suarez et al., 2020; Sudhakara et al., 2018).

It is thus reasonable to consider the probability, that pre‐existing periodontal disease may have a negative impact on COVID‐19 infection, in terms of further exacerbation of the systemic inflammatory response. Indeed, in a case−control study using patient hospital records, a significant association between periodontal disease and severe COVID‐19‐related outcomes was observed. Specifically, 82.5% of patients with severe COVID‐19 symptomatology were also diagnosed with Stage II, III, and IV periodontitis, while periodontitis patients had nine times higher chances to die from COVID‐19, compared to non‐periodontitis patients (Marouf et al., 2021). Alternatively, it can be hypothesized that patients that develop severe COVID‐19 infection are a population with a tendency for disproportional inflammatory response to an otherwise mild trigger. Based on this particular immune reaction, it is probable that these patients may also present more severe periodontal destruction at an earlier age, compared to the general population. If so, severe periodontitis could serve as a screening criterion at hospital admission, indicating patients with a higher risk for severe COVID‐19.

The present pilot study aimed to assess whether younger patients (≤60 years) that have been hospitalized in ICU for severe COVID‐19 infection were susceptible to severe periodontitis.

## 2 METHODS

### 2.1 Study population

The study was part of the study protocol of the COVID‐19‐Database of the University Hospital of Geneva, approved by the Human Ethics Committee from the canton of Geneva, Switzerland (BASEC #: 2020‐00917); the study was conducted in accordance with the Helsinki Declaration as revised in 2013.

Patients ≤60 years of age diagnosed with COVID‐19 and hospitalized in the ICU in the University Hospitals of Geneva during the first two waves of the global pandemic (April 2020−June 2021) were selected for the purpose of this study.

The patients’ contact information was obtained from the Medical Records Department of the hospital and patients were contacted by telephone regarding their willingness to participate in the study. Patients already receiving dental treatment in private practice were asked to fill a consent form permitting to contact their dentist, to obtain the most recent (less than a year old) radiographs. For patients without a dentist, a free visit for a full clinical and radiographic examination at the Division of Regenerative Dental Medicine and Periodontology of the University Clinics of Dental Medicine of the University of Geneva, was proposed. A single experienced examiner (P. G.) performed the clinical and radiographic examination. Periodontal probing depths, bleeding on probing, furcation involvement, and recessions were recorded at six sites per tooth of every tooth present and a full status of periapical radiographs was taken.

### 2.2 Study design

Susceptibility to periodontitis was assessed by using radiographic bone loss (RBL) as a surrogate measurement, similarly to many epidemiological studies (Farook et al., 2020; Pitiphat et al., 2004), since not all patients underwent a full‐mouth clinical examination. Any type of 2D dental radiographs, that is, posterior bitewings, panoramic and periapical radiographs, were used; when multiple types of 2D radiographs were available, periapicals were selected due to higher accuracy (Reed & Polson, 1984).

**Figure 1:**
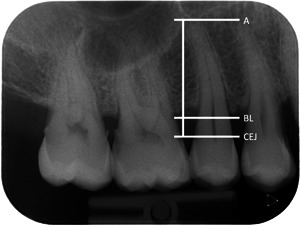
An example of RBL measurement in a periapical radiograph. RBL was calculated as the distance from the CEJ to the most coronal level of the alveolar crest (BL), where the periodontal ligament appeared with normal width. RBL was expressed as a percentage (%) of the total length of the root of the tooth, that is, CEJ to apex (A). BL, bone level; CEJ, cemento‐enamel junction; RBL, radiographic bone loss.

Interproximal RBL was measured using as reference the cemento‐enamel junction (CEJ) to the most coronal crestal bone level (BL), where the periodontal ligament appeared with a normal width, at the most affected aspect of the most affected tooth on the radiographs. RBL was expressed as a percentage (%) of the total length of the root of the tooth (CEJ to apex) (Figure 1). The radiographs were assessed using dedicated medical screens and low‐light conditions. On bitewing radiographs, 1 mm was calculated as equal to 10% bone loss, as proposed by Lang and Tonetti for the periodontal risk assessment (Lang & Tonetti, 2003). After excluding teeth with local causes that could have provoked bone loss, such as caries, periodontal‐endodontic lesions, cracked and fractured roots, other restorative reasons, or mispositioned third molars, periodontitis on the patient level was defined as bone loss at two or more nonadjacent teeth. The most affected tooth was used for the calculation of RBL, since it represents the severity of the periodontal destruction, as suggested in the recent classification of periodontal and peri‐implant diseases (Papapanou et al., 2018). Based on RBL as the only measure, the patients were classified into: Stage I if RBL was less than 15%, Stage II if RBL was 15%−33%, and Stage III/IV if RBL was more than 33%. The grade was defined based also on the current classification using as the only parameter the RBL to age ratio on the most severely affected tooth. Patients were Grade A if the ratio was less than 0.25, Grade B if it was between 0.25 and 1, and Grade C if it was more than 1. High susceptibility to periodontitis was defined as being Stage III/IV, Grade C periodontitis (Tonetti et al., 2018).

General anamnestic information regarding the systemic disease, medication, and smoking habits was also collected and/or extracted from the medical records.

Examiner reliability was assessed by measuring 29 randomly selected radiographs among 15 patients who were not part of the study by two independent experienced examiners (P. G., A. Z.) at two different time points. Intra‐class correlation coefficients for RBL in % presented a kappa index of 0.92.

### 2.3 Statistical analysis

Descriptive statistics were used for data presentation using SPSS, version 20.0.

## 3 RESULTS

Of the 315 patients hospitalized in the ICU with COVID‐19 diagnosis, 141 patients were hospitalized during the first wave and 174 during the second wave. Ninety patients passed away. Of the remaining 225 patients, 136 were excluded because of their age (>60 years old). Of the 89 remaining patients, only 32 accepted to participate. The other 57 patients, either refused to participate or had residual pathology and difficulties in mobility, or they were highly occupied or unreachable. Of the 32 participants, 2 were completely edentulous and thus excluded, resulting in a final number of 30 eligible patients that were included in the study. Finally, 20 of those patients did not have a regular dentist, so they were offered and accepted to attend a full‐mouth periodontal examination at the University Clinics of Dental Medicine.

Table 1 displays the general characteristics of the study population. The mean age of the participants was approximately 50 years old with more than half of them (60%) being equal to or more than 50 years old. None of the participants was a smoker and only three of them (10%) were former smokers. Only one of the participants had diabetes Type II but was well‐controlled with a low hemoglobin A1c (HBA1c) test (glycated hemoglobin test), less than 6%. Three patients (10%) presented hypertension (HTA), with 2 (6.6%) of them being diagnosed with cardiovascular disease (CVD). Other comorbidities were hypercholesterolemia (HCA) at 10% of the patients, hyperthyroidism, prostate inflammation, asthma, osteoporosis, migraines, human immunodeficiency virus (HIV) infection, and allergies with a prevalence of 3.3% each. Depression was noted for two of the participants (6.6%).

**Table 1:** General characteristics of each of the included subjects (*n* = 30)

Age (mean 49.5)	Gender (22M− 8F)	Smoking (0)	Diabetes (3.3%)	Comorbidities
58	M	N	N	HCA
58	M	N	N	HTA
59	M	N	N	N
52	F	N	N	N
59	M	N	N	Hyperthyroidism, prostate inflammation
51	M	Ex	N	HTA, HCA
39	M	N	N	Asthma
50	M	N	N	N
49	M	Ex	N	HCA
49	M	N	N	N
28	M	N	N	N
50	M	N	N	HIV, CVD
60	F	N	N	Osteoporosis, depression
56	F	N	N	HTA, CVD
38	F	N	N	N
41	F	Ex	N	Depression
55	M	N	N	Migraines
38	M	N	N	N
60	F	N	N	N
55	M	N	N	N
55	M	N	N	N
39	F	N	N	N
59	M	N	N	N
25	M	N	N	N
46	M	N	N	N
57	M	N	N	N
43	M	N	N	N
37	M	N	N	N
59	M	N	N	N
59	F	N	Y	Several allergies

Abbreviations: CVD, cardiovascular disease; Ex, former; F, female; HCA, hypercholesterolemia; HTA, hypertension; M, male; N, no, yes.

The percentage of RBL of the most severely affected tooth is presented in Table 2. Out of the 30 patients, 9 (30%) presented RBL up to 15%, 7 (23.3%) up to 33%, and 14 (46.7%) more than 33% RBL.

**Table 2:** Bone loss (%) assessed in panoramic, bitewings or periapical radiographs

Radiographic bone loss (RBL)	0%−15%	15%−33%	More than 33%
Panoramic radiograph (OPT)			
Number of patients (%)	1 (3.3%)	—	2 (6.7%)
Bite‐wing (BWs)			
Number of patients	2 (6.7%)	—	1 (3.3%)
Periapical radiographs			
Number of patients	6 (20%)	7 (23.3%)	11 (36.7%)
All radiographs			
Number of patients	9 (30%)	7 (23.3%)	14 (46.7%)

All participating patients were periodontally compromised (Table 3). Approximately half of them presented with a mild to moderate extent of periodontal destruction, with a low to medium risk for progression (Stages I and II and Grades A and B). The other half showed advanced defects and moderate to high risk (Stages III and IV and Grades B and C). The prevalence of Stage III/IV, Grade C periodontitis was estimated to be 26.67%. The diagnosis made on the basis of the radiographs was actually confirmed by the clinical examination in all of the 20 attending patients.

**Table 3:** Periodontal diagnosis for each of the included subjects, based on radiographic assessment.

Age (mean 49.5)	Gender (22M−8F)	Diagnosis: stage and grade
58	M	II ‐ B
58	M	I ‐ A
59	M	IV ‐ C
52	F	III ‐ B
59	M	IV ‐ C
51	M	IV ‐ C
39	M	I ‐ A
50	M	II ‐ B
49	M	III ‐ B
49	M	II ‐ B
28	M	I ‐ A
50	M	I ‐ A
60	F	I ‐ A
56	F	IV ‐ B
38	F	I ‐ A
41	F	IV ‐ C
55	M	II ‐ B
38	M	II ‐ B
60	F	III ‐ C
55	M	III ‐ C
55	M	I ‐ A
39	F	IV ‐ C
59	M	II ‐ B
25	M	II ‐ A
46	M	I ‐ A
57	M	IV ‐ B
43	M	III ‐ C
37	M	I ‐ A
59	M	III ‐ B
59	F	IV ‐ B
Total		
Stage I Grade A&B	9 (30%)	
Stage II Grade A&B	7 (23.33%)	
Stage III&IV Grade B	6 (20%)	
Stage III&IV Grade C	8 (26.67%)	

## 4 DISCUSSION

The results of this pilot study, involving a limited number of relatively young patients (≤60 years) that have been hospitalized in the ICU of the University Hospital of Geneva due to COVID‐19 infection, showed that about 27% were severely periodontally compromised and had a high risk for disease progression (i.e., periodontitis Stage III/IV, Grade C). These percentages are somehow in accordance with percentages reported in previous studies. For example, in a cross‐sectional study, 82 patients who were tested positive for COVID‐19 were included and had a clinical examination and serological evaluation of blood parameters related to COVID‐19 infection. It was reported that 20.7% of patients with Stage III and 13.4% of the patients with Stage IV periodontitis were hospitalized (Gupta et al., 2022). In the same study, for non‐periodontitis or gingivitis patients, the hospital admission rates were respectively 13.4% and 11%. In another case−control study, using a database of patient records to assess periodontal conditions as risk factors for COVID‐19, a significant association between periodontal disease and COVID‐19‐related outcomes was observed. Specifically, it was shown that 82.5% of the patients with severe COVID‐19 symptomatology were also diagnosed with Stage II, III, and IV periodontitis, while patients with periodontitis had nine times higher chances to die from COVID‐19 compared to periodontally healthy individuals (Marouf et al., 2021).

In the present study, subjects older than 60 years were excluded. This fact reduced the number of patients eligible but also reduced confounding factors, such as the influence of older age and associated comorbidities, contributing to the severity of COVID‐19 (Mueller et al., 2020) and to the severity of periodontal disease (Genco, 1996; Grossi et al., 1994, 1995). In our study, the diagnosis of periodontitis was based only on radiographic assessment, to include also patients that were unable to come for a clinical examination. Furthermore, taking into account radioprotection concepts (United Nations Scientific Committee on the Effects of Atomic Radiation UNSCEAR, 2008), all types of available radiographs less than a year old were included, and no new ones were taken; thus, in a very few patients, panoramic radiographs were included, something that might compromise the precision of the radiographic measurements. Nevertheless, in all 20 patients that received a clinical examination (2/3 of the patients), diagnosis based on the radiographic assessment—including those few cases with panoramic radiographs—was confirmed by the diagnosis based on the clinical status. All these patients presented active periodontal disease. Thus, the findings of the present study seem to corroborate the notion that periodontal disease may have a negative impact on COVID‐19 infection, in terms of further exacerbating the systemic inflammatory response.

As already mentioned, another hypothesis is that patients developing severe COVID‐19 symptoms represent a population with a tendency for disproportional inflammatory response to an otherwise relatively mild trigger; such patients may have experienced more severe periodontal destruction at an earlier age, compared to the general population. In a recent publication, from a European population, the prevalence of periodontitis Stage III is 21.7% and Stage IV seems to be around 1% for those under 60 years old (Stodle et al., 2021). Herein, the fraction of patients with periodontitis Stage III/IV was about double. Specifically, 47% of the participants were classified as periodontitis Stage III/IV and about 27% of the participants were even classified to have a high susceptibility to periodontitis (Stage III/IV, Grade C). This finding seems to support the above hypothesis of a hyperresponsive fraction of the population, and it seems reasonable to suggest that it should be further investigated.

Obviously, a major limitation of the present study is the very small number of included patients. Unfortunately, a large number of patients simply did not agree to participate in the study, while others could not participate due to residual pathology, fatigue, and mobility problems. As shown also in a study by Crook et al. in 2021, patients hospitalized in ICU, but not only, often present a range of persistent symptoms after infection (Crook et al., 2021). This condition, now identified as “long COVID,” is recognized by many research institutes. According to Zarei et al. (2021), long COVID consists of signs and symptoms that appear during, or after a SARS‐CoV‐2 infection and last longer than 12 weeks, and are not related to another diagnosis (Zarei et al., 2021). Similarly, to acute COVID‐19, long COVID can affect various organs and systems, such as the respiratory, cardiovascular, neurological, gastrointestinal, and musculoskeletal systems. Its most common symptoms include fatigue, dyspnea, cardiac abnormalities, cognitive impairment, sleep disturbances, muscle pain, concentration problems, and headaches, making patients vulnerable and restricting them from their daily habits. That seemed also to be a complaint of many of the patients that we contacted. Another limitation of the present study is the lack of a control group including COVID‐19 patients matched for age and sex, but without ICU hospitalization history.

Within the limitations of this pilot study, it may be suggested that periodontitis patients and highly susceptible periodontitis patients have a high risk to develop severe forms of COVID‐19, including IUC hospitalization. The hypothesis that periodontitis diagnosed by dental radiographs can be used as a screening tool for predicting severe COVID‐19 disease needs further investigation in larger scale epidemiological studies.

## AUTHOR CONTRIBUTIONS

Catherine Giannopoulou and Andreas Stavropoulos contributed to the conception, design of the study and drafted and critically revised the manuscript. Panagiotis Gardelis performed the clinical and radiographic examination, contributed to the analysis and interpretation of the data, drafted and critically revised the manuscript. Alkisti Zekeridou performed the radiographic analysis,contributed to the analysis and interpretation of the data, drafted and critically revised the manuscript. Noemie Suh and Christophe Le Terrier provided the participants and critically revised the manuscript.

## CONFLICT OF INTEREST

The authors declare no conflict of interest.

## Data Availability

The data that support the findings of this study are openly available.
